# E-cigarette use and susceptibility among Indonesian youth: the role of social environment, social media, and individual factors

**DOI:** 10.1186/s12889-025-24013-3

**Published:** 2025-08-14

**Authors:** Mouhamad Bigwanto, Melinda Pénzes, Nurul Kodriati, Emma Rachmawati, Nida Amalia, Róbert Urbán

**Affiliations:** 1 Doctoral School of Psychology, Eötvös Loránd University, Izabella u. 46, Budapest, 1064 Hungary; 2 Institute of Psychology, Eötvös Loránd University, Izabella u. 46, Budapest, 1064 Hungary; 3 Faculty of Health Sciences, Universitas Muhammadiyah Prof. Dr. HAMKA, Jl. Limau II, Jakarta, 12210 Indonesia; 4 Data-Driven Health Division of the National Laboratory for Health Security, Health Services Management Training Centre, Semmelweis University, Kútvölgyi út 2, Budapest, H-1125 Hungary; 5 Faculty of Public Health, University of Ahmad Dahlan, Jl. Prof. DR. Soepomo SH, Yogyakarta, 55164 Indonesia; 6 School of Public Health, Georgia State University, Atlanta, GA 30303 US; 7 Faculty of Public Health, Universitas Muhammadiyah Kalimantan Timur, Jl. Ir. H. Juanda No. 15, Samarinda, 75124 Indonesia

**Keywords:** Electronic cigarettes, Social environment, Social media marketing, Youth, Student, Indonesia

## Abstract

**Background:**

Youth behavior is significantly influenced by their social environment and social media (SM). Susceptibility to e-cigarette use, defined as the likelihood of initiating e-cigarette use among non-users, is a critical early marker for prevention efforts. This study explores the interplay of social environment, SM marketing exposure, and individual traits in e-cigarette use and susceptibility among Indonesian youth, addressing a gap in non-Western contexts.

**Methods:**

A school-based online survey of 1,600 Indonesian youth aged 15–24, conducted from March to August 2023 in Jakarta, Yogyakarta and East Kalimantan, assessed e-cigarette use, susceptibility, and predictors, including social environment, SM marketing exposure, and sensation-seeking behavior. Structural equation modeling and multinomial regression were used to analyze associations.

**Results:**

Approximately 13.3% of students reported using e-cigarettes in the past 30 days, and 6.7–10.1% of non-users were susceptible to experimenting with e-cigarettes. Boys were significantly more likely than girls to be current (OR = 6.67, 95% CI [3.05–14.57]) and ever e-cigarette users (OR = 2.92, 95% CI [2.10–4.06]). Sensation-seeking (OR = 2.19, 95% CI [1.83–2.62]), e-cigarette use by horizontal family member (OR = 1.39, 95% CI [1.10–1.53]), number of friends using e-cigarettes (OR = 1.20, 95% CI [1.17–1.23]), and exposure to e-cigarette advertisements on Instagram and TikTok (OR = 1.35, 95% CI [1.20–1.53]) were significant predictors of current use. Among non-users, boys reported higher susceptibility to e-cigarette use than girls (β = 0.20, *p* <.01). Sensation seeking (β = 0.24, *p* <.01) and the number of friends using e-cigarettes (β = 0.22, *p* <.01) were directly associated with susceptibility. Sex-specific patterns were observed: maternal and sisters’ e-cigarette use were associated with increased susceptibility among girls (*r* =.11, *p* =.002; *r* =.17, *p* <.001), while grandfathers’ use was linked to higher susceptibility among boys (*r* =.21, *p* <.001). Notably, TikTok exposure was uniquely associated with greater susceptibility among girls (*r* =.08, *p* =.023).

**Conclusions:**

SM, peers, and family significantly shape youth e-cigarette-related behavior. These findings underscore the need for targeted interventions, including banning e-cigarette advertising on SM, promoting peer-driven prevention strategies, and leveraging SM for educational campaigns to reduce youth e-cigarette use.

**Supplementary Information:**

The online version contains supplementary material available at 10.1186/s12889-025-24013-3.

## Background

Electronic cigarettes or e-cigarettes entered the global market around 2007 [1]. Emerging evidence suggests that e-cigarette use (or vaping) is associated with various health issues, including cardiovascular diseases (such as arrhythmia, hypertension, and endothelial dysfunction), as well as adverse effects on the respiratory and central nervous systems, and oral health [2–8]. Furthermore, research on the effectiveness of e-cigarette as a smoking cessation aid remains inconclusive [9]. While some studies indicate that e-cigarettes may assist smokers in quitting [10, 11] others do not support that conclusion [12] and highlight their role in promoting initiation, particularly among youth [13].

The uncertainty surrounding e-cigarettes as a smoking cessation aid has been intensified by the emergence of a paradox: increasing e-cigarette use among adolescents worldwide [14, 15]. A systematic review and meta-analysis conducted in 2021 found that approximately 17.2% of children and adolescents aged 8–19 years in 69 countries reported ever having used e-cigarettes, while 7.8% were classified as current users (those who had used e-cigarettes in the past 30 days) [16]. One possible factor contributing to these high rates is the aggressive marketing of e-cigarettes to young people by tobacco companies [17]. As reported in the Indonesian National Health Survey, the prevalence of e-cigarette use among youth aged 10–18 years increased from 0.06% in 2018 to 0.13% in 2023, highlighting a worrying upward trend [18, 19]. Without government intervention, this figure is likely to increase. Effective measures could include banning the use of e-cigarettes in public places, and restricting advertising and promotion on different media platforms to create a social environment that discourages youth’s use [20]. Since their legalization through taxation by the Ministry of Finance in 2018 [21], e-cigarettes have been freely promoted and marketed in Indonesia with no restrictions until 2024. These products are widely available, not only in specialized vape shops, but also in small convenience stores. They come in a variety of appealing shapes and flavors, often designed to attract young consumers. In addition, vape stores often work with other businesses, such as restaurants and hairdressers, to promote e-cigarettes through joint marketing campaigns [22].

The rise of e-cigarette use can be approached through two analytical approaches that explore the factors influencing e-cigarette use and their interactions: the host-agent-environment-vector epidemiological framework [23], also known as the epidemiologic triangle, and Bronfenbrenner’s social-ecological model. 1) The epidemiological framework identifies characteristics of the host, such as age, gender, genetic predisposition, sensory novelty seeking, curiosity, and more. For example, e-cigarette use has increased more rapidly among younger adults compared to other age groups [24], while sensation seeking has consistently emerged as a significant predictor of adolescent experimentation and use [25]. The agent refers to the characteristics of e-cigarettes, including their sensory appeal (attractive design, exciting and varied flavors), composition (especially the addictive properties of nicotine), and accessibility. For example, flavors, particularly fruit and menthol/mint, play a critical role in attracting users to e-cigarettes, with fruit flavors serving as a strong motivator for experimentation among young adults [26]. The environment encompasses many factors, including the immediate social surroundings, the influence of the online environment, and broader aspects such as legal regulations. For example, exposure to peer vaping or media advertising of e-cigarettes may normalize their use among US adolescents [27]. Finally, the vector includes the tobacco industry, its marketing strategies, such as online advertising [28, 29], and the characteristics of retail outlets selling e-cigarettes [22, 30]. The interaction between host, agent, environment, and vector remains a complex area that has not been fully explored, highlighting the need for further research to understand its implications on behaviors such as e-cigarette use. (2) On the other hand, Bronfenbrenner’s social-ecological model, widely used in public health to understand health behavior issues, includes multiple levels of influence on individual behavior. These include personal factors (such as gender, age, interests, and education), relationships with family, school personnel, peers, and health professionals (microsystem), interactions between these microsystems (mesosystem), influences from local politics and mass media (exosystem), and broader influences such as national policies and cultural attitudes (macrosystem) [31]. Both approaches emphasize that understanding e-cigarette use requires a complex and multidimensional analysis that considers interactions across multiple levels and factors.

Building on these frameworks, it is critical to consider the unique role of social media as a powerful vector in the modern information ecosystem, particularly in shaping behaviors and perceptions related to e-cigarette use. The advent of the Internet has transformed the mass media landscape, enabled instant global communication, and fostered the rise of social media platforms. According to Meltwater’s 2024 Digital Report for Indonesia, Instagram has a monthly usage rate of 85.3%, followed by Facebook at 81.6% and TikTok at 73.5% [32]. These platforms have influenced traditional advertising and information dissemination strategies, including those used by the tobacco industry to promote e-cigarettes [33]. Instagram, TikTok, and Facebook have become popular places for e-cigarette advertising. A report from Grand View Research highlights that e-cigarettes are often promoted online [34] through visually appealing content that portrays them as fashionable lifestyle accessories. Focus group studies in Scotland found that 11- to 16-year-olds identified Instagram and TikTok as the main sources of e-cigarettes. Influencers on these platforms often portray e-cigarettes as ‘cool’ and fashionable lifestyle accessories [35]. Similarly, in Indonesia, e-cigarette promotions frequently feature influencers with a predominantly young audience, with 58% of Instagram posts promoting e-cigarettes featuring lifestyle-related content [29].

Digital media creates significant loopholes in tobacco control regulations, particularly in tobacco advertising, promotion, and sponsorship (TAPS). Unlike traditional advertising platforms, digital media often bypasses restrictions, allowing tobacco companies to target younger audiences through social media campaigns, influencer partnerships, and other innovative strategies. This complicates enforcement efforts and underscores the urgent need for updated regulations [36].

As of January 2023, Indonesia ranked fourth globally in internet usage, following China, India, and the United States, with over 200 million users [37]. Among Indonesian youth aged 9–19, an overwhelming 93.52% actively engage with social media [38], highlighting the integral role that these platforms play in the daily lives of teenagers.

The primary objective of this study is to investigate the influence of peer and family e-cigarette use, social media marketing exposure, and individual characteristics such as sensation-seeking behavior and demographic factors (e.g., age, sex, school type, and location) on both current e-cigarette use and susceptibility among Indonesian youth. It also aims to provide insights into how these factors interact within a cultural and regulatory context distinct from those of Western countries, where much of the existing research has been conducted.

We hypothesized that individual traits such as sensation-seeking behavior and demographic factors, including age, sex, school type, and location, significantly impact both current e-cigarette use and susceptibility. We also hypothesized that the immediate social environment significantly influences both current e-cigarette use and susceptibility, but the effects are not uniform. Specifically, the influence of peers and siblings (horizontal family members) is stronger than that of parents or grandparents. Lastly, we hypothesized that social media marketing exposure significantly influences both current e-cigarette use and susceptibility.

## Method

### Participants and procedure

A school-based, online cross-sectional survey was conducted among youth aged 15–24 years from March to August 2023 in three Indonesian provinces with the highest prevalence of e-cigarette use. A more detailed description of the sampling and study procedure has been presented elsewhere [26]. Briefly, based on population statistics, sample sizes of 385 respondents for Jakarta and 384 for Yogyakarta and East Kalimantan were required for a 95% confidence level. Students from randomly selected private and public high schools (two private, two public) and universities (one private, one public) in each province participated in the study. After contacting school and university principals, the questionnaire was distributed in classrooms using the Qualtrics platform. Only specific classrooms were approached, with three classrooms selected from each school or university. For universities, three first-year classrooms were selected, while for schools, one classroom from each grade (grades 1, 2, and 3) was chosen. The online questionnaire from Qualtrics was completed through two methods: via students’ personal smartphones and, in schools equipped with computer laboratory facilities, through school computers. Of the 1,799 respondents approached, complete responses with informed consent to participate were obtained from 1,600 participants, resulting in an 88.9% response rate. Incentives in the form of phone credit valued at IDR 50,000 (approximately USD 3) were awarded to 30 randomly selected respondents to encourage participation. Written informed consent was obtained from each participant, and for high school students, additional consent was obtained from their respective classroom teachers.

### Measures

This study utilized a detailed questionnaire to collect data from participants. In this report, we used six key areas: 1) the social environment, including e-cigarette use by peers and family members, 2) exposure to e-cigarette marketing on social media, 3) sensation-seeking behavior, 4) demographic details (sex, age, school type, and school location), 5) susceptibility to smoking and e-cigarette use, and 6) smoking and e-cigarette use status.

The sections on sensation-seeking traits, susceptibility to e-cigarette use, and e-cigarette use status identification were adapted from validated instruments previously used in related studies, with detailed references provided in each section below. As the original items were in English, a validation process was undertaken to ensure linguistic and cultural appropriateness. First, a forward and backward translation (English-Bahasa) was conducted by two English-speaking Indonesian natives to ensure accuracy. Second, cognitive interviews were conducted with two high school students and two first-year university students. Feedback from these processes was used to refine and finalize the questionnaire before field data collection. The list of questions for the present study is provided in Supplementary File 1.

#### Perceived social environment: e-cigarette use of peers and family members

Respondents were asked to report the e-cigarette use of their close family members: grandfather, grandmother, father, mother, brother, and sister (response options: yes/no). Additionally, they indicated how many of their close friends currently use e-cigarettes, with response options ranging from “none (0)” to “nine or more (9)”. For e-cigarette use among close family members, we applied Principal Component Analysis (PCA) to reduce variables into uncorrelated components, simplifying data while retaining most variability for concise and interpretable results. Two components were retained: (1) e-cigarette use by vertical family members (father, mother, grandfather, and grandmother), explaining 25.9% of the variance, and (2) e-cigarette use by horizontal family members (sisters and brothers), explaining 43.0% of the variance.

#### E-cigarette marketing exposure on social media

Respondents were asked if they had ever seen ads or promotions for e-cigarettes on social media. Those who responded ‘yes’ identified the platforms on which they encountered these ads, with options including Instagram, YouTube, Facebook, Twitter, TikTok, Line, and others. Principal component analysis was used to create composite scores representing e-cigarette ad exposure across social media platforms. Two components were retained: The first component, representing ad exposure on Instagram and TikTok, explained 33.5% of the variance, while the second component, capturing exposure on other social media platforms, accounted for 49.8% of the variance. Higher scores on these composite measures indicate stronger exposure to e-cigarette ads on the respective social media platforms.

#### Sensation seeking behaviors

Sensation seeking was measured using the 8-item Brief Sensation Seeking Scale (BSSS-8), referred to as SSS and covering experience seeking, thrill and adventure seeking, disinhibition, and boredom susceptibility [39, 40]. Respondents rated items on a five-point scale ranging from “strongly disagree” to “strongly agree”. The total sensation-seeking score was calculated using the average item scores, showing satisfactory internal consistency (α = 0.81).

#### Demographic information

Students’ age (continuous variable), sex (male or female), the type of institution (private or public), and the location of their school or university (rural or urban) were included in the analyses. Based on administrative divisions, the three provinces comprise a total of 9 cities (*kota*) and 12 districts (*kabupaten*). For the purpose of analysis, *kabupaten* were classified as rural areas, while *kota* were categorized as urban areas.

#### Susceptibility to e-cigarette use

Three items adapted from previous studies [41, 42] were used to measure susceptibility or intention to use e-cigarettes among participants who had not yet tried them. For e-cigarettes, respondents were asked whether they planned to try them in the future, whether they would try them within the next year, and whether they would accept one if offered by their best friend. The response options were definitely not (1), probably not (2), probably yes (3), and definitely yes (4). Principal component analysis was used to construct separate susceptibility scores for e-cigarettes. The three questions were grouped into a single principal component which explained 84.5% of the variance. Higher scores on the composite susceptibility measure reflect stronger susceptibility to e-cigarette use among never users.

#### E-cigarette use

Participants were asked two questions to assess their use of e-cigarettes. The first question asked if they had ever tried e-cigarettes, even just one or two puffs, with response options of “yes” or “no”. Those who answered ‘yes’ but had not used e-cigarettes in the past 30 days were classified as ever users, while those who had used them in the past 30 days were classified as current users, and non-users were individuals who had never used e-cigarettes [43].

### Statistical analysis

Descriptive statistics and multinomial regression were conducted using *SPSS* (version 25) [44], while the structural equation model (SEM) was performed in *Mplus* 8.10 [45]. Pearson correlations were used to initially analyze variables related to e-cigarette use, social media marketing exposure, and e-cigarette use among family and friends, with the results stratified by sex. To further explore sex differences, the tested correlations were analyzed in greater detail.

To investigate factors influencing susceptibility to e-cigarette use among non-users, we conducted a path analysis within a SEM framework. In this path analysis, the variables were categorized into distal and proximal groups, with mediation effects tested for the proximal variables. Distal variables included sex, age, school characteristics (type and location), the personality trait of sensation-seeking, and horizontal family use. The proximal variables, serving as mediators, were the number of friends using e-cigarettes and exposure to e-cigarette advertising on Instagram and TikTok. The outcome variable was susceptibility to e-cigarette use. Variables that did not show significant correlations with other variables in the correlation analysis were excluded from the model. The model was estimated using Maximum Likelihood (ML) estimation with bootstrapping (*n* = 1000) to obtain robust standard errors for parameter estimates. Model fit indices (e.g., RMSEA, CFI, TLI) were assessed to ensure acceptable model fit.

Lastly, multinomial regression analyses were conducted to identify predictors of current and ever e-cigarette use. In the first analysis, non-users served as the reference group, enabling comparisons between current users, ever users, and non-users. In the second analysis, ever users were set as the reference group to examine differences between current and ever users.

To account for the clustered nature of the data, standard errors were adjusted for the school region using the TYPE = COMPLEX procedure in Mplus version 8.10. This adjustment was successfully applied in the multinomial logistic regression model. However, in the case of the full path analysis model, clustering correction could not be implemented due to model complexity and the limited number of clusters. To evaluate the potential impact of clustering, we first calculated intracluster correlation coefficients (ICCs) for all study variables. Based on these values, we computed the design effect (DEFF) using the standard formula: DEFF = 1 + (n̄– 1) × ρ, where n̄ is the average cluster size and ρ is the ICC [46]. DEFF values were below the commonly used threshold of 2 for all variables except age (see in the Supplementary File 2, Supplementary Table 1), suggesting that clustering was unlikely to introduce major bias in the estimates [47]. Given these results and the technical limitations, the path model was estimated without clustering correction.

## Result

### Descriptive statistics

Table 1 provides descriptive statistics on e-cigarette use, friends and family members using e-cigarettes, and exposure to e-cigarette marketing on social media, categorized by sex. Detailed analyses of variables such as age, sensation-seeking scores, and school/university location and type by sex are reported in a previous article [26].

**Table 1:** Descriptive statistics of e-cigarette marketing exposure, family and peer influence, and e-cigarette use by sex

Characteristics	Total Sample	Sex		
	*N* = 1600	Girls	Boys	χ^2^/t (*p*-value)
E-cigarette use *N (%)*				
Non-users*	1010 (63.4)	696 (74.7)	263 (44.1)	160.4 (< 0.001)
Ever users	371 (23.2)	172 (18.5)	192 (32.2)	
Current user**	211 (13.2)	64 (6.9)	142 (23.8)	
Family members currently using e-cigarettes *(Yes) N (%)*				
Mother	9 (0.6)	7 (0.7)	2 (0.3)	1.09 (0.496)
Father	122 (7.9)	73 (7.8)	49 (8.1)	0.05 (0.814)
Grandmother	10 (0.7)	5 (0.5)	5 (0.8)	0.49 (0.526)
Grandfather	24 (1.6)	9 (1.0)	15 (2.5)	5.57 (0.018)
Sister	44 (2.9)	33 (3.5)	11 (1.8)	3.81 (0.051)
Brother	464 (30.2)	293 (31.3)	171 (28.4)	1.49 (0.222)
Number of friends using e-cigarette, *Mean (SD)*	4.93 (3.42)	4.59 (3.43)	5.49 (3.35)	t = 4.98 (< 0.001)
Exposure to e-cigarette marketing in Social Media platforms *(Yes) N (%)*				
Instagram	799 (52.0)	513 (54.9)	286 (47.5)	7.94 (0.005)
TikTok	568 (37.0)	390 (41.7)	178 (29.6)	23.17 (< 0.001)
YouTube	380 (24.7)	225 (24.1)	155 (25.7)	0.55 (0.455)
Facebook	225 (14.6)	128 (13.7)	97 (16.1)	1.72 (0.190)
X/Twitter	219 (14.2)	170 (18.2)	49 (8.1)	30.22 (< 0.001)
Line	40 (2.6)	31 (3.3)	9 (1.5)	4.78 (0.029)
Others	217 (14.1)	125 (13.4)	92 (15.3)	1.10 (0.293)
E-cigarette use in the family (indices)				
Vertical family use (parents, grandparents)^$^	0.00 (1.00)	−0.02 (0.92)	0.03 (1.1)	1.06 (0.291)
Horizontal family use (sister, brothers)^$^	0.00 (1.00)	0.04 (1.04)	−0.03 (0.95)	1.22 (0.222)
Social Media use indices				
Instagram & TikTok ads exposure^$^	0.00 (1.00)	0.09 (1.01)	−0.13 (0.97)	4.30 (< 0.001)
Other social media ads exposure^$^	0.00 (1.00)	0.01 (0.93)	0.00 (0.93)	0.13 (0.900)
Personality				
Sensation seeking, *Mean (SD)*	2.53 (0.74)	2.48 (0.71)	2.63 (0.77)	3.99 (< 0.001)

*Never used the products

**Used the products within the last 30 days

^$^Principal component score

Boys were significantly more likely than girls to be both current e-cigarette users (OR = 5.87, 95% CI [4.23–8.15]) and ever users (OR = 2.95, 95% CI [2.30–3.79]). Regarding family members using e-cigarettes, boys were more likely to have a grandfather who uses e-cigarettes (OR = 2.63, 95% CI [1.14–6.04]). Significant sex differences were also observed in the number of friends using e-cigarettes, with boys reporting higher numbers than girls (Cohen’s *d* = 0.27, 95% CI [0.16–0.37]). Regarding social media exposure, girls were more likely than boys to encounter e-cigarette advertisements on Instagram (OR = 1.34, 95% CI [1.09–1.64]), TikTok (OR = 1.70, 95% CI [1.37–2.12]), and X/Twitter (OR = 2.51, 95% CI [1.79–3.51]). Composite scores for social media use also indicated higher exposure to Instagram and TikTok advertisements among girls compared to boys (Cohen’s *d* = 0.23, 95% CI [0.12–0.33]).

### Determinants of susceptibility to e-cigarette use among non-users

When examining item-level response frequencies, clear patterns emerge regarding the likelihood of trying e-cigarettes. Approximately 6.7–10.1% of non-users reported a likelihood of trying e-cigarettes. Although only a small fraction (0.1–0.2%) selected “definitely yes,” a notably larger proportion (6.7–9.9%) responded “probably yes” when asked about experimenting with e-cigarettes (Supplementary File 2, Supplementary Table 2). The correlation analyses presented in Table 2 reveal that e-cigarette susceptibility was significantly and positively associated with the number of friends using e-cigarettes and sensation-seeking behavior. Sex differences were notable, with boys exhibiting higher susceptibility to future e-cigarette use compared to girls. Sensation-seeking behavior also showed significant associations with both the number of friends using e-cigarettes and exposure to Instagram and TikTok advertisements, emphasizing its central role in shaping e-cigarette-related attitudes and behaviors.

**Table 2:** Correlation matrix of variables associated with e-cigarette use susceptibility among non-users with e-cigarettes

	1	2	3	4	5	6	7	8	9	10
1. E-cigarette use susceptibility^$^										
2. Age	.05									
3. Sex	.20**	-.13**								
4. School/University Type	.06	−.08*	−.06							
5. School/University Location	−.01	.19**	.07^#^	.03						
6. No. of Friends using e-cigarette	.22**	.09**	.10**	−.00	−.01					
7. E-cigarette use in the family– Vertical (parents, grandparents)^$^	−.02	−.04	.06	−.03	.05	−.01				
8. E-cigarette use in the family– Horizontal (sister, brothers)^$^	.05	−.05	.00	.01	−.01	.11**	.03			
9. Instagram & TikTok ads exposure^$^	.04	−.03	-.15**	.06	.06	.15**	−.01	.09**		
10. Other social media ads exposure^$^	.06	−.06	.02	-.11**	.04	.06	.03	.05	.20**	
11. Sensation seeking	.24**	−.02	−.00	−.04	.03	.16**	−.01	.04	.11**	.07*

*N* = 840. #: *N* = 890. *p <.05; **p <.01. $: Principal Component Score (mean = 0.00; SD = 1.00). Sex is coded as 1 = girls and 2 = boys; School/University Type is coded as 1 = private and 2 = public; School/University Location is coded as 1 = rural and 2 = urban

*p <.05; **p <.01. Boldfaced correlations remained significant after Bonferroni correction (*p* <.0045)

Sex differences in the tested correlations were further analyzed, with detailed results presented in Supplementary File 2, Supplementary Table 3. These analyses showed that the association between the number of friends using e-cigarettes and susceptibility to e-cigarette use was significantly stronger among boys (*r* =.29, *p* <.001) than girls (*r* =.17, *p* <.001; z = 1.78, *p* =.038). Interestingly, maternal and sister’s e-cigarette use were significantly associated with higher susceptibility only among girls (*r* =.11, *p* =.002 and *r* =.17, *p* <.001, respectively), while these associations were not significant for boys. In contrast, grandfather’s e-cigarette use was significantly associated with susceptibility only among boys (*r* =.21, *p* <.001).

For exposure to e-cigarette advertisements, TikTok exposure was significantly associated with susceptibility among girls (*r* =.08, *p* =.023). Additionally, exposure to e-cigarette ads on Twitter was significantly associated with susceptibility in both girls (*r* =.15, *p* <.001) and boys (*r* =.15, *p* =.013).

**Fig. 1:**
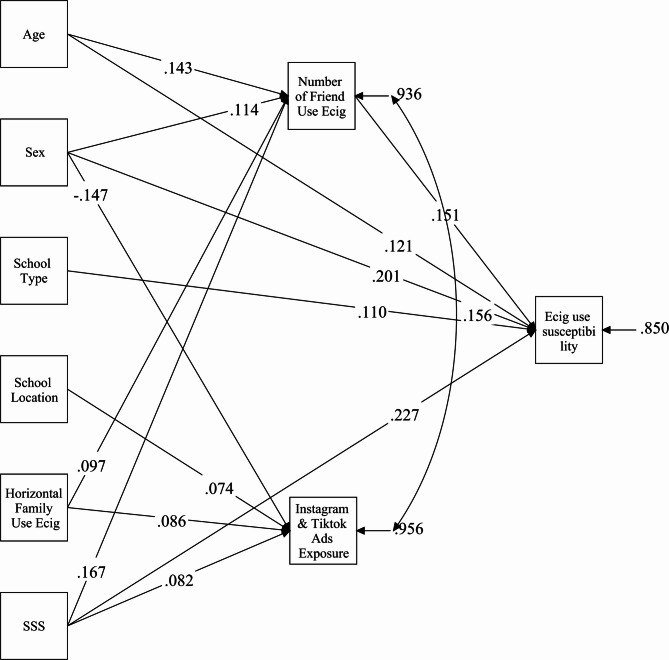
Path analysis model depicting relationships between socio-demographic variables, sensation-seeking, horizontal family members’ e-cigarette use, friends’ e-cigarette use, social media exposure, and susceptibility to e-cigarette use. Note: Only significant path coefficients (*p* <.05) are presented, and all coefficients are standardized. Sex is coded as 1 = girls and 2 = boys; School/University Type is coded as 1 = private and 2 = public; School/University Location is coded as 1 = rural and 2 = urban. All continuous variables were scaled from low to high. SSS = Sensation Seeking Score

We estimated a path model to explore the relationships between predictors and e-cigarette use susceptibility. The model is presented in Fig. 1. The model fit was acceptable (χ² = 3.0, df = 5, *p* =.5494; CFI = 1.00; TLI = 1.00; RMSEA = 0.000 [0.000–0.042]; SRMR = 0.010). Age, being male, having a higher number of friends using e-cigarettes, enrollment in a public school, and higher sensation-seeking scores were significant direct predictors of susceptibility. In contrast, exposure to social media advertisements and horizontal family member’s use were not associated with susceptibility.

The number of friends using e-cigarettes was positively predicted by age, being male, having horizontal family member who use e-cigarettes, and higher sensation-seeking scores. Exposure to ads on Instagram and TikTok was predicted by being female, having horizontal family member who use e-cigarettes, and higher sensation-seeking scores.

We also examined indirect effects through mediation analysis. We identified three significant mediations: (1) The path SSS → the number of friends using e-cigarettes → susceptibility was significant (*β* = 0.025 *p* =.002) and explained 19% of the association between sensation seeking and susceptibility; (2) The path being a male → number of friends using e-cigarettes → susceptibility was also significant (*β* = 0.013 *p* =.020) and explained 7% of the association between sex and susceptibility; (3) The path age → the number of friends using e-cigarettes → susceptibility was also significant (*β* = 0.016 *p* =.005) and explained 18% of the association between age and susceptibility.

### Predictors of e-cigarette use: social environment and social media advertising exposure

Multinomial regression analyses were conducted to identify predictors of current and ever e-cigarette use. The first analysis used non-users as the reference group to compare current and ever users with non-users. The second analysis used ever users as the reference group to compare current users with ever users. The results were summarized in Table 3.

**Table 3:** Multinomial regression analysis of predictors for current and ever e-cigarette use

	Ever use# OR [95% CI]	Current use# OR [95% CI]	Current use (vs. ever use) ## OR [95% CI]
1. Age	0.99 [0.93–1.06]	1.07 [1.00-1.14]	1.07 [0.98–1.18]
2. Sex	2.92 [2.10–4.06]***	6.67 [3.05–14.57]***	2.28 [1.34–3.89]***
3. School/University Type	1.06 [0.83–1.34]	1.52 [0.79–2.93]	0.62 [0.36–1.08]
4. School/University Location	0.75 [0.59–0.95]*	0.99 [0.70–1.41]	1.33 [0.92–1.91]
5. No. of Friends using e-cigarette	1.09 [1.05–1.13]***	1.20 [1.17–1.23]***	1.10 [1.06–1.15]***
6. E-cigarette use in the family– Vertical (parents, grandparents)^$^	0.93 [0.83–1.04]	1.06 [0.99–1.13]	1.14 [1.00-1.30]
7. E-cigarette use in the family– Horizontal (sister, brothers)^$^	1.28 [1.10–1.49]**	1.39 [1.10–1.77]**	1.09 [0.93–1.27]
8. Instagram & TikTok ads exposure^$^	1.10 [0.96–1.27]	1.35 [1.20–1.53]***	1.23 [1.13–1.34]***
9. Other social media ads exposure^$^	0.98 [0.90–1.06]	0.86 [0.67–1.11]	0.88 [0.67–1.16]
10. Sensation seeking	1.66 [1.44–1.91]***	2.19 [1.83–2.62]***	1.31 [1.05–1.64]*

*N* = 1349, Standard errors are adjusted for clustering at the school region level. #: Reference group is never users. ##: Reference group is ever users

Sex is coded as 1 = girls and 2 = boys; School/University Type is coded as 1 = private and 2 = public; School/University Location is coded as 1 = rural and 2 = urban.

*p <.05; **p <.01. $: Principal Component Score (mean = 0.00; SD = 1.00). Odds ratios with *p* values less than 0.01 are highlighted in bold

#### Current e-cigarette use vs. non-use

Several factors strongly predicted current e-cigarette use compared to non-use. Boys were significantly more likely to report current use than girls. Public school students had higher odds of current use compared to private school students. Peer and family influences played a key role, with a higher number of friends who used e-cigarettes significantly increasing the odds of current use. Having a horizontal family member that used e-cigarettes was also a significant predictor. Exposure to advertising on Instagram and TikTok was associated with increased odds of being a current user. Sensation-seeking behavior was a strong predictor, with higher scores significantly increasing the likelihood of current use.

#### Ever e-cigarette use vs. non-use

Significant predictors of ever e-cigarette use compared to non-use were also identified. Boys were more likely to be ever users than girls. The number of friends who used e-cigarettes was positively associated with ever use, with each additional friend increasing the odds. Similarly, having horizontal family members who used e-cigarettes significantly predicted ever use. Sensation-seeking behavior was strongly associated with ever use, with higher scores increasing the likelihood of being an ever user.

#### Current e-cigarette use vs. ever use

Several predictors remained significant when comparing current users with ever users. Boys had higher odds of being current users than girls. Public school students were more likely to be current users than private school students. The number of friends who used e-cigarettes was a significant predictor of current use compared to ever use. Exposure to advertising on Instagram and TikTok was also significantly associated with current use relative to ever use. Sensation-seeking behavior remained a significant factor, with higher scores moderately increasing the odds of being a current user compared to an ever user.

## Discussion

This study provides unique insights into the factors influencing e-cigarette use and susceptibility among Indonesian youth, a population largely understudied in the global discourse on tobacco control. Indonesia, with its high prevalence of male smokers and rapidly growing e-cigarette market, represents a critical setting for understanding how social, individual, and environmental factors interact to shape youth behavior in a non-Western context.

Indonesia has the highest prevalence of male smokers globally, with nearly two-thirds of Indonesian men identified as smokers [48], highlighting an alarmingly high rate of tobacco use among men. This widespread behavior has normalized smoking among men, making it more socially acceptable compared to women. Similarly, this study found a parallel trend in e-cigarette use among youth, with boys demonstrating significantly higher e-cigarette use rates than girls. Even among non-users, boys were more susceptible to e-cigarette use compared to girls. Using tobacco products, in any form, appears to be socially acceptable for males in Indonesia. Among men, tobacco use is deeply ingrained in social norms [49], and e-cigarettes are increasingly normalized among young males, as they are often associated with a modern lifestyle [22, 50]. Further research is essential to validate these observations and to examine the underlying cultural and social factors contributing to the normalization of e-cigarettes among young males.

Sensation-seeking behavior in the context of e-cigarette use is highly relevant, particularly among youth who exhibit high sensation-seeking tendencies and are more likely to engage in risky behaviors, including experimenting with substances like e-cigarettes [51, 52]. Research suggests that sensation seekers are often drawn to the novelty and perceived excitement of e-cigarettes, including features like diverse flavor combinations, which make these emerging products particularly appealing to this group [26]. Furthermore, similar to conventional smoking [52], high sensation-seeking youth also report having more friends who use e-cigarettes, reinforcing their engagement in such behaviors through peer influence. Our findings confirm this effect in the context of e-cigarette use, highlighting the interplay between sensation-seeking traits and social networks in shaping youth behavior. In addition, our study found that sensation-seeking youth are more aware of e-cigarette advertisements, particularly on platforms like TikTok and Instagram. It remains unclear whether this heightened awareness is due to paying greater attention to these advertisements or simply having a stronger memory of them. Regardless, this increased awareness likely amplifies the influence of social media marketing on their behavior, making this group particularly susceptible to targeted advertising strategies.

Our study confirmed the hypothesis that the social environment, particularly peers and horizontal family members, plays a pivotal role in influencing e-cigarette use behavior among youth (both current and ever users). At this developmental stage, peers and horizontal family members exert a greater influence on behavior compared to parents [53]. The more peers and horizontal family members use e-cigarettes, the more likely it is that youth perceive this behavior as normative, facilitating its uptake [54, 55]. Moreover, peers and horizontal family members may also increase youth access to e-cigarettes, either by sharing these products directly or by serving as sources of information about their availability and appeal.

Interestingly, our findings revealed sex-specific patterns. Among girls, maternal and sisters’ e-cigarette use was significantly associated with higher susceptibility to e-cigarette use, whereas these associations were not significant among boys. Conversely, among boys, grandfathers’ e-cigarette use was significantly associated with increased susceptibility, and boys were more likely to have a grandfather who uses e-cigarettes. This association may point to a broader generational influence. We hypothesized that grandfathers might use e-cigarettes as a tool for smoking cessation, leading to their presence in the household. This visibility could attract young boys’ attention, normalizing e-cigarette use within the family environment, a phenomenon we propose to call the ‘grandfather effect.’ Similarly, grandmothers’ or mothers’e-cigarette use might exert comparable influence on girls. These dynamics could represent a form of ‘vertical exposure,’ distinct from direct social learning pathways. Further research is needed to test these hypotheses and explore the role of intergenerational influence on youth susceptibility to e-cigarette use.

Our study also identified significant sex differences in the number of friends using e-cigarettes, with boys reporting higher number of friends use e-cigarette than girls. This creates an additional supportive environment for boys to initiate e-cigarette use. Research consistently demonstrates that youth with friends who use tobacco or e-cigarettes are more likely to start using these products themselves, as such behaviors become normalized within their social circles [52–55]. Therefore, public health strategies should incorporate peer-based approaches in tobacco prevention programs. These programs should aim to disrupt cycles of influence by promoting tobacco-free norms and delivering education on the harms of tobacco and nicotine products through peer-to-peer interventions.

Exposure to e-cigarette advertisements on Instagram and TikTok was significantly associated with current e-cigarette use but did not predict susceptibility to e-cigarette use among non-users. A previous study highlights the role of social media in influencing e-cigarette use among youth [56]. However, the question remains: why is exposure to e-cigarette advertisements on social media not associated with susceptibility among non-users? This discrepancy may be because these individuals are either uninterested in the content, rendering them immune to the promotions, or because they are not actively targeted. Social media advertisements often operate as tailored or “tuned” advertising, with algorithms optimizing ad delivery based on user engagement. Consequently, individuals who express interest in e-cigarette products are exposed to more advertisements and promotions, while non-users, who lack such interest, encounter significantly fewer advertisements [57]. Another possibility is that non-users already have a high awareness of the harmful effects of these products [58, 59], information that is readily available on social media alongside promotional content. Another alternative explanation for our findings is that e-cigarette users may have a stronger recall of advertisements than non-users, suggesting that these ads might reinforce existing behaviors rather than initiate new ones. This exposure could help sustain and normalize e-cigarette use, serving as a tool for maintaining engagement rather than motivating first-time use. Further research is needed to confirm this hypothesis and clarify whether advertising primarily sustains use patterns or drives initiation.

A closer examination, however, revealed notable sex-specific differences. Among girls, exposure to TikTok was significantly associated with higher susceptibility to e-cigarette use, indicating the unique influence of this platform in shaping their attitudes and behaviors. These findings underscored the complex role of social media in influencing youth behavior and highlighted the importance of understanding platform-specific and gendered advertising strategies. A study on e-cigarette marketing on Instagram in Indonesia revealed that 64% of feed posts promoting e-cigarettes on selected accounts featured images of women. This strategy is not only effective in attracting male engagement but also contributes to normalizing e-cigarette use among women [29]. Such targeted marketing strategies emphasize the need for public health interventions that address the specific ways e-cigarette companies use gendered imagery to influence youth behavior.

In general, the environment, including peers, horizontal family members, and social media, plays a multifaceted role in influencing tobacco use among youth. Social media has the potential to both negatively and positively affect e-cigarette use behavior among adolescents. Individual factors such as sex and sensation-seeking traits are inherent and unlikely to change. However, the surrounding environment, including marketing and peer influence, can be modified to positively shape youth behavior.

Currently, Government Regulation No. 28 of 2024 as a derivative of Health Law No. 17 of 2023, had banned the advertisement, promotion and sponsorship of tobacco products including e-cigarettes on social media in Indonesia [60]. Advertising and promotion of e-cigarettes in retail stores also regulated by this Regulation. However, the success of the implementation will depend largely on the technical procedures regulation that the Indonesian government will establish. Therefore, to support the implementation, it is essential for policymakers to have reliable information on how social environment, social media, and individual factors relate to e-cigarette use among youth.

### Study limitations

The cross-sectional design of this study severely limits the ability to draw causal inferences. While we assume that explanatory variables, such as demographic and personality factors, precede and may predict behavior, the possibility of reverse causality or the influence of unmeasured third variables cannot be completely ruled out. Furthermore, as pointed out by Maxwell and Cole (2007) [61], performing mediation or path analysis on cross-sectional data can introduce bias in estimating indirect and direct effects. Without longitudinal control, mediation effects may be either underestimated or overestimated due to model misspecification and the absence of temporal ordering. While our model is grounded in theory and based on continuous variables, which supports the validity of our analytic approach, we acknowledge that future longitudinal studies are needed to confirm the hypothesized mediation pathways and strengthen causal interpretation. Although our model includes only continuous or quasi-continuous variables, meeting the assumptions of traditional path analysis, future studies may benefit from applying formal causal mediation techniques, which are based on the potential outcomes framework and allow for more flexible effect definitions and modeling of interactions [62].

The study sample was drawn from three Indonesian provinces (Jakarta, Yogyakarta, and East Kalimantan) with the highest prevalence of e-cigarette use. These provinces differ substantially in terms of urbanization, socio-economic characteristics, and cultural context. Jakarta being a highly urbanized metropolis, Yogyakarta a mid-sized academic and cultural hub, and East Kalimantan a resource-rich, less urbanize province. These contextual differences may influence vaping-related behaviors and limit the generalizability of our findings to other regions in Indonesia. Nevertheless, the inclusion of these diverse settings, along with the unique cultural and regulatory context of Indonesia, provides valuable insights into e-cigarette use patterns in middle-income Southeast Asian countries.

Data collection relied on self-reported responses, which introduces potential sources of bias. Recall bias may affect the accuracy with which participants report past behaviors, while social desirability bias may lead to underreporting of behaviors perceived as stigmatized, such as e-cigarette use. To mitigate these risks, we ensured full anonymity and did not collect any identifying information (e.g., school name or exact location). Additionally, survey items were carefully worded in a neutral, non-judgmental tone, and the questionnaire was self-administered without the presence of an interviewer, reducing potential response pressure. Despite these precautions, the possibility of reporting bias cannot be completely ruled out and should be taken into account when interpreting the findings.

## Conclusions

Along with individual factors such as sex and sensation-seeking traits, our findings underscore the significant influence of peers, horizontal family members, and the pervasive role of social media in shaping youth behaviors related to e-cigarette use. Implementing strategies such as banning e-cigarette advertisements and promotions on social media, involving youth in peer-driven prevention initiatives, and leveraging social media to disseminate prevention campaigns can be pivotal in reducing youth vaping behaviors. Effective public health interventions must address these social determinants and proactively counter the harmful effects of e-cigarette marketing and promotions on social media to decrease e-cigarette use among youth.

Longitudinal studies with representative samples are necessary to examine the temporal sequence and causal relationships between personal factors (such as sex, age, and sensation-seeking traits), family and peer relationships (microsystem), media influences (exosystem), and broader factors such as national policies and cultural attitudes (macrosystem) in relation to e-cigarette use among youth.

## Supplementary Information

Supplementary Material 1.

Supplementary Material 2.

## Data Availability

The datasets used and analyzed in this study are available from the corresponding author upon reasonable request.

## Abbreviations

E-Cigarette: Electronic cigarette
PCA: Principal component analysis
SEM: Structural equation model
SSS: Sensation seeking scale
