# Supplementary material for: E-cigarette use and susceptibility among Indonesian youth: the role of social environment, social media, and individual factors
Source: BMC Public Health. 2025 Aug 14;25:2756. doi: 10.1186/s12889-025-24013-3 (PMC12351980; doi:10.1186/s12889-025-24013-3)
Supplement: Supplementary file 1 — Supplementary Material 1. [file 12889_2025_24013_MOESM1_ESM.docx]

**Questionnaire**

*(Please fill in or put a check mark* ***√***  *in the answer choices****)***

**Sociodemographic**

1. How old are you?

(e.g. = pretend you are 14 years and your birthday is in 2 weeks, then you should answer 14 years. You become 15 years only from the day of your 15th birthday).

1. What is your sex? Boy Girl Other, not want to specify
2. The type of educational institution you are attending now?

a. Private high school b. Public high school c. Private University d. Public University

**E-cigarette use** *(e-cigarette is a nicotine delivery product that uses a battery from the electronic device to heat up a liquid with nicotine into an aerosol that users inhale)*

1. [Have you ever tried vaping/using an e-cigarette even one or two puff](https://doi.org/10.1515/ijamh-2019-0172)?

a. No, never b. Yes

1. [During the past 30 days, how many days did you use e-cigarettes?](https://doi.org/10.1515/ijamh-2019-0172) *(Please answer 0 if you answer never tried vaping/using e-cigarettes in the previous question or you didn’t smoke cigarette in the past 30 days)*

a. 0 b. 1 - 15 days c. 16-29 days d. Every day for the past 30 days

**Peers and family member use e-cigarette**

1. How many of your close friends currently using e-cigarettes?

None b. 1 c. 2 d. 3 e. 4 f. 5 g. 6 h. 7 i. 8 j. 9 or more

1. Please checklist if any of your close family members below currently using e-cigarettes?

Father Mother Sister Brother Grandmother Grandfather

None

**For non-user only:**

1. Do you think that in the future you might experiment with e-cigarettes?

a. Definitely not b. Probably not c. Probably yes d. Definitely yes

1. At any time during the next year do you think you will use e-cigarette?

a. Definitely not b. Probably not c. Probably yes d. Definitely yes

1. If one of your best friends were to offer you e-cigarette, would you use it?

a. Definitely not b. Probably not c. Probably yes d. Definitely yes

**Exposure to e-cig marketing and promotion**

1. Have you ever seen ads or promotions for e-cigarettes on social media:

Yes No

If yes, specify which one, there can be more than one choice:

❑ Instagram ❑ Youtube ❑ Facebook ❑ Twitter/X
❑ Tiktok  ❑ LINE  ❑ Others (.............................)

Now we are also interested in your general interest. Please record the appropriate answer for each item, depending on whether you Strongly agree, agree, disagree, or strongly disagree with it. 1 = Strongly disagree, 2 = Disagree, 3 = Neither disagree or agree, 4 = Agree, 5 = Strongly Agree.

_____ I would like to explore strange places
_____ I get restless when I spend too much time at home
_____ I like to do frightening things
_____ I like wild parties
_____ I would like to take off on a trip with no pre-planned routes or timetables

_____ I prefer friends who are excitingly unpredictable
_____ I would like to try bungee jumping
_____ I would love to have new and exciting experiences, even if they are illegal
