# Supplementary material for: E-cigarette use and susceptibility among Indonesian youth: the role of social environment, social media, and individual factors
Source: BMC Public Health. 2025 Aug 14;25:2756. doi: 10.1186/s12889-025-24013-3 (PMC12351980; doi:10.1186/s12889-025-24013-3)
Supplement: Supplementary file 2 — Supplementary Material 2. [file 12889_2025_24013_MOESM2_ESM.docx]

**Supplementary File 2.**

*Supplementary Table 1.* Intracluster correlation (ICC) and design effect

| Variables | Intracluster correlation (rho) | Design effect (DEFF) |
| --- | --- | --- |
| No. of Friends using e-cigarette | 0.008 | 1.664 |
| Vertical family use (Parents, grandparents)^$^ | 0.007 | 1.581 |
| Horizontal family use (sister, brothers)^$^ | 0.006 | 1.498 |
| Instagram & TikTok ads exposure^$^ | 0.011 | 1.913 |
| Other social media ads exposure^$^ | 0.019 | 2.577 |
| Intention to use e-cigarette | 0.006 | 1.498 |
| Sensation seeking | 0.012 | 1.996 |
| Age | 0.317 | 27.311 |

N=1,596. The average cluster size (n) is 84. Cluster variable: Study_Location.

DEFF=1+rho(n-1).

*Supplementary Table 2*. Percentage of susceptibility to e-cigarette use among non-users (N=1,010)

|  | definitely not n (%) | probably not n (%) | probably yes n (%) | definitely yes n (%) |
| --- | --- | --- | --- | --- |
| Do you think that in the future you might experiment with e-cigarettes? | 732 (73.6) | 162 (16.3) | 98 (9.9) | 2 (0.2) |
| At any time during the next year do you think you will use e-cigarette? | 761 (76.6) | 165 (16.6) | 67 (6.7) | 1 (0.1) |
| If one of your best friends were to offer you e-cigarette, would you use it? | 769 (77.4) | 140 (14.1) | 83 (8.4) | 2 (0.2) |

*Supplementary Table 3.* Correlations of susceptibility to e-cigarette use among non-users, by sex

|  | Girls  r (*p*)  *n*=696 | Boys  r (*p*)  *n*=263 | z (*p*) |
| --- | --- | --- | --- |
| Father e-cigarette use | -.01 (.870) | .09 (.160) | 1.28 (.099) |
| Mother e-cigarette use | **.12** (.002) | .01 (.834) | -1.45 (.073) |
| Sister e-cigarette use | **.17** (<.001) | .01 (.942) | -2.30 (.011) |
| Brother e-cigarette use | -.06 (.131) | .11 (.083) | 0.68 (.246) |
| Grandmother e-cigarette use | -.04 (.300) | -.02 (.753) | -0.81 (.206) |
| Grandfather e-cigarette use | .07 (.076) | **.22** (<.001) | 2.09 (.018) |
| No. of Friends using e-cigarette | **.17** (<.001) | **.29** (<.001) | 1.78 (.038) |
| Ads exposure |  |  |  |
| Instagram | .04 (.222) | .11 (.073) | 0.87 (.190) |
| Youtube | -.01 (.628) | -.06 (.308) | -0.61 (.269) |
| Facebook | -.05 (.124) | .06 (.291) | 1.70 (.044) |
| Twitter | **.15** (<.001) | **.15** (.013) | 0.00 (.500) |
| TikTok | **.08** (.023) | .04 (.483) | -0.58 (.278) |
| Line | -.03 (.303) | -.03 (.595) | -0.98 (.163) |
| Others | -.02 (.522) | -.11 (.062) | 1.26 (.103) |
